# A novel approach for biomarker selection and the integration of repeated measures experiments from two assays

**DOI:** 10.1186/1471-2105-13-325

**Published:** 2012-12-06

**Authors:** Benoit Liquet, Kim-Anh Lê Cao, Hakim Hocini, Rodolphe Thiébaut

**Affiliations:** 1 Univ. Bordeaux, ISPED, centre INSERM U-897-Epidémiologie-Biostatistique, Bordeaux, F-33000, FRANCE; 2 INSERM, ISPED, centre INSERM U-897-Epidémiologie-Biostatistique, Bordeaux, F-33000, FRANCE; 3 Queensland Facility for Advanced Bioinformatics and the institute for Molecular Bioscience, The University of Queensland, Brisbane, QLD 4072, Australia; 4 INSERM U955 Eq 16, UPEC Université, Créteil, FRANCE; 5 Vaccine Research Institute ANRS, Paris, France

## Abstract

**Background:**

High throughput ’omics’ experiments are usually designed to compare changes observed between different conditions (or interventions) and to identify biomarkers capable of characterizing each condition. We consider the complex structure of repeated measurements from different assays where different conditions are applied on the same subjects.

**Results:**

We propose a two-step analysis combining a multilevel approach and a multivariate approach to reveal separately the effects of conditions within subjects from the biological variation between subjects. The approach is extended to two-factor designs and to the integration of two matched data sets. It allows internal variable selection to highlight genes able to discriminate the net condition effect within subjects. A simulation study was performed to demonstrate the good performance of the multilevel multivariate approach compared to a classical multivariate method. The multilevel multivariate approach outperformed the classical multivariate approach with respect to the classification error rate and the selection of relevant genes. The approach was applied to an HIV-vaccine trial evaluating the response with gene expression and cytokine secretion. The discriminant multilevel analysis selected a relevant subset of genes while the integrative multilevel analysis highlighted clusters of genes and cytokines that were highly correlated across the samples.

**Conclusions:**

Our combined multilevel multivariate approach may help in finding signatures of vaccine effect and allows for a better understanding of immunological mechanisms activated by the intervention. The integrative analysis revealed clusters of genes, that were associated with cytokine secretion. These clusters can be seen as gene signatures to predict future cytokine response. The approach is implemented in the R package mixOmics (http://cran.r-project.org/) with associated tutorials to perform the analysis^a^.

## Background

Recent advances in high throughput ‘omics’ technologies enable quantitative measurements of expression or abundance of biological molecules in a whole biological system. Various popular omics platforms in systems biology include transcriptomics, proteomics, cytomics and metabolomics. These experiments are usually designed to compare changes observed between different conditions or groups and are often used to identify biomarkers capable of characterising pathological states or response to treatment.

The decreasing costs of these high-throughput platforms now enable repeated measures experiments on the same individuals or biological samples. Such experiments allow a substantial gain in information. For instance, longitudinal designs are more powerful as they reduce the noise due to inter individual variability, as long as the correlation between repeated observations is taken into account. There exists an abundant literature on the analysis of repeated measurements of omics data [1,2]. In this context, a common approach is to apply a univariate mixed model on each gene followed by multiple testing correction [3]. However, this approach disregards the dependency between genes, and due to the high dimensionality of the data, numerous hypotheses tests must be performed.

The mixed model approach has been used for the analysis of one single data type (e.g. gene expression). However, a growing number of high-throughput data are generated in standard clinical trials. For example, the evaluation of HIV vaccine in phase I/II trials incorporates measurements of counts of numerous types of cell, of the production of intra and extracellular cytokines and of gene expression [4]. The integration of such multi-layer information can help unravel the complexities of a biological system, as each functional level is hypothesized to be related to each other [5]. However the integration of omics data is a challenging task. Firstly, the large number of measured biological entities makes it very difficult to obtain a good overview or understanding of the system under study. Secondly, the small number of samples or patients makes statistical inference difficult and argue for using the maximum amount of available information. Thirdly, the integration of heterogeneous data represents an analytical and numerical challenge when trying to find common patterns in data from different origins.

In recent years, several multivariate approaches have been proposed to combine two omics data, often in an unsupervised framework. In contrast to univariate repeated measures analysis, these linear multivariate approaches take into account the dependency between genes, are able to handle large and noisy data sets and do not face computational issues in the high dimensional case as matrix inversions are avoided. Most importantly in the context of this study, they enable the integration of data coming from different platforms and provide interpretable visualisation tools. These approaches aim at selecting correlated biological entities from two [6-11] or more data sets [12]. In particular, with sparse Partial Least Squares (sPLS) we have shown that the integrative analysis of large scale omics datasets could generate new knowledge not accessible by the analysis of a single data type alone [7,8]. The biological relevance of this approach has been illustrated recently in some studies [13,14].

The flexibility and versatility of PLS also enable a supervised framework through PLS-Discriminant Analysis (PLS-DA [15]). A variant of which has recently been proposed to select discriminative features that best separate the different conditions (sPLS-DA, [16]). sPLS-DA was shown to give similar performances to classical classification methods such as Machine Learning approaches and variants of Linear Discriminant Analysis and was recently applied in a biological study [17].

In this paper, we consider a two-step approach to model the correlation between repeated measurements while taking advantage of the multivariate approaches. We first propose to extract the within-sample variation [18-20] before analysing this transformed data set using sPLS-DA for a discriminant analysis or sPLS for an integrative analysis.

Starting from the classical mixed-model, we present the principle of a multilevel analysis to extract the within-sample deviation of the data and we extend the approach to a two-factor analysis. The within data set is then analysed with either sPLS-DA to select discriminative genes between the groups of subjects on a single data set, or with sPLS to select subsets of correlated variables from two data sets. A simulation study is performed which demonstrates the good performance of multilevel sPLS-DA compared to a classical sPLS-DA. The approach is then illustrated on an HIV vaccination study, where the effect of a lipopeptide based vaccine was explored by measuring before and after vaccination various components of the immune response, including gene expression and cytokine secretion. These repeated measurement were made in several *in vitro* conditions on Peripheral Blood Mononuclear Cells: ‘NS’ (no stimulation); HIV Gag peptides ‘GAG+’ (peptides included in the vaccine), HIV Gag peptides ‘GAG-’ (peptides not included in the vaccine) and ‘LIPO5’ (all five peptides included in the vaccine).

## Methods

### Notations

Let ***X***(*N *× *p*) and ***Z***(*N* × *q*) represent two data matrices (e.g. gene expression and cytokine secretion). We denote by *N* the total number of samples (or rows) in the data, and by *n* the number of experimental units (or unique subjects), *p* (resp. *q*) is the total number of genes (resp. cytokines), also called variables or predictors. The dummy matrix ***Y***(*N *× *G*) indicates the group/treatment of the samples, with *G* the total number of groups.

### Multilevel approach

We first present the mixed-effect model as a pedagogical tool and then introduce the concept of the multilevel approach based on the “split-up” variation. Despite the fact that some similarities exist between the mixed-effect model and the multilevel “split-up” variation approach, we emphasize that the latter is performed completely independently from the estimation of the mixed-effect model. Moreover, the mixed model relies on certain assumptions (such as Gaussian distribution of random effects) that the split-up variation approach does not require.

#### Mixed-effect model

Let $\left(X_{\mathrm{sj}}^{k}\right)$ be the gene expression of a given gene *k* for subject *s* with stimulation *j*. In this context, the mixed model is defined by:

$$\begin{array}{lll} X_{\mathrm{sj}}^{k}\; & =\;\mu_{j}^{k}+\pi_{s}^{k}+\varepsilon_{\mathrm{sj}}^{k},\;s=1,\ldots ,n,\;j=1,\ldots ,G_{s}\;\\ \; & =\;\mu_{\cdot \cdot }^{k}+\alpha_{j}^{k}+\pi_{s}^{k}+\varepsilon_{\mathrm{sj}}^{k}\; & \; \end{array}$$

where for a given gene *k*, $\left(\mu_{j}^{k}\right)$ measures the fixed effect of stimulation *j*, which can be further decomposed into $\left(\mu_{\cdot \cdot }^{k}\right)$, the overall mean stimulation effect, plus $\left(\alpha_{j}^{k}\right)$ which is the differential effect for stimulation *j*. The $\left(\pi_{s}^{k}\right)$ are independent random variables following a normal distribution $\left(N(0,\sigma_{\pi ,k}^{2})\right)$, which take into account the dependency between the repeated measures made on the same subject *s*, the residuals $\left(\varepsilon_{\mathrm{sj}}^{k}\right)$ are independent random variables following a $\left(N(0,\sigma_{\varepsilon ,k}^{2})\right)$ distribution. Note that the number of stimulation for each subject (*G*_*s*_) may differ. However, for each subject *s* we observe no more than one observation for each stimulation *j*, thus the subject effect interactions with the stimulation factor are confounded with the residuals. We also assume that $\left(\pi_{s}^{k}\right)$ and $\left(\varepsilon_{\mathrm{sj}}^{k}\right)$ are independent.

This model is known also as the one-way unbalanced random-effects ANOVA. A simple approach for identifying differentially expressed (DE) genes in this model is to test the stimulation effect for each gene and apply a multiple testing correction (FDR from [21] set to 5%). Following this global test, pairwise comparison can then be applied between two stimulations, followed by multiple correction (e.g. FDR, 5%). Some limitations of this standard approach are discussed in the Results and discussion Section. The main advantage of the mixed model is the introduction of the random element $\left(\pi_{s}^{k}\right)$, which is specific to the subject *s* and represents the between-subject deviation. In the same spirit, the multilevel approach based on the split-up variation focusses on separating the different sources of variation: the within-subject deviation (“variation”) and the between-subject deviation.

#### Split-up variation

As suggested by Westerhuis et al. [19] in the mixed model framework, the observation $\left(X_{\mathrm{sj}}^{k}\right)$ can be decomposed into:

(1) $$x_{\mathrm{sj}}^{k}=\underset{\text{offset}}{\underset{︸}{x_{\cdot \cdot }^{k}}}+\underset{\text{between-subject deviation}}{\underset{︸}{\left(x_{s\cdot }^{k}-x_{\cdot \cdot }^{k}\right)}}+\underset{\text{within-subject deviation}}{\underset{︸}{\left(x_{\mathrm{sj}}^{k}-x_{s\cdot }^{k}\right)}}$$

where $\left(x_{\cdot \cdot }^{k}=\frac{1}{N}\overset{G_{s}}{\underset{j=1}{\sum }}\overset{n}{\underset{s=1}{\sum }}x_{\mathrm{sj}}^{k}\right)$ and $\left(x_{s\cdot }^{k}=\frac{1}{G_{s}}\overset{G_{s}}{\underset{j=1}{\sum }}x_{\mathrm{sj}}^{k}\right)$. The offset term $\left(x_{\cdot \cdot }^{k}\right)$ is an estimation of $\left(\mu_{\cdot \cdot }^{k}\right)$, the between-subject deviation is an estimation of $\left(\pi_{s}^{k}\right)$ and the within-subject deviation is an estimation of $\left(\alpha_{j}^{k}+\varepsilon_{\mathrm{sj}}^{k}\right)$ and can be further decomposed as:

$$\underset{\text{within-subject deviation}}{\underset{︸}{\left(x_{\mathrm{sj}}^{k}-x_{s\cdot }^{k}\right)}}=\underset{\text{Stimulation effect}}{\underset{︸}{\left(x_{\cdot j}^{k}-x_{\cdot \cdot }^{k}\right)}}+\underset{\text{residual}}{\underset{︸}{\left(x_{\mathrm{sj}}^{k}-x_{s\cdot }^{k}-x_{\cdot j}^{k}+x_{\cdot \cdot }^{k}\right)}}$$

where $\left(x_{\cdot j}^{k}=\frac{1}{n_{j}}\overset{n_{j}}{\underset{s=1}{\sum }}x_{\mathrm{sj}}^{k}\right)$, with *n*_*j*_ the number of subject undergoing stimulation *j*. *Therefore, a part of the within-subject deviation is explained by the stimulation effect.*

Let ***X*** be the (*N*× *p*) gene expression matrix on *s*=1,…,*n* subjects with *G*_*s*_ stimulations (in the balanced case *N*=*n*× *G*, otherwise $\left(N=\overset{n}{\underset{s=1}{\sum }}G_{s}\right)$). According to equation (1):

$$X=\underset{\text{offset term}}{\underset{︸}{X_{\cdot \cdot }}}+\underset{\text{between-subject deviation}}{\underset{︸}{X_{b}}}+\underset{\text{within-subject deviation}}{\underset{︸}{X_{w}}}$$

The matrix ***X***_··_ represents the offset term defined as $\left(1_{\text{N}}x_{\cdot \cdot }^{T}\right)$, where ***1***_N_ is the (*N*× 1) matrix containing ones and $\left(x_{\cdot \cdot }^{T}=(x_{\cdot \cdot }^{1},\ldots ,x_{\cdot \cdot }^{p})\right)$; ***X***_***b***_ is the between-subject matrix of size (*N*× *p*) defined by concatenating $\left(1_{G_{s}}x_{bs}^{T}\right)$ for each subject into ***X***_***b***_ with $\left(x_{\mathrm{bs}}^{T}=(x_{s\cdot }^{1}-x_{\cdot \cdot }^{1},\ldots ,x_{s\cdot }^{p}-x_{\cdot \cdot }^{p})\right)$; ***X***_***w***_ =***X***−***X***_*s*·_ is the within-subject matrix of size (*N*× *p*), with ***X***_*s*·_ the matrix defined by concatenating the matrices $\left(1_{{\text{G}}_{s}}x_{s\cdot }^{T}\right)$ for each subject into ***X***_***s***·_, with $\left(x_{s\cdot }^{T}=(x_{s\cdot }^{1},\ldots ,x_{s\cdot }^{p})\right)$.

Similarly to the Analysis of Variance, it is easy to show that the sum of squares can be separated into three parts:

(2) $$||X|{|}^{2}=||X_{\cdot \cdot }|{|}^{2}+||X_{b}|{|}^{2}+||X_{w}|{|}^{2},$$

where ||***X***||^2^=*trace*(***X***^*T*^***X***). Equation (2) can be used to evaluate the magnitude of the different sources of variation.

The mixed-model described earlier can provide an analysis for repeated measurements data in an unbalanced design. It can be viewed as an extension of a paired t-test to test the differences between paired observations. However, to tackle some of the previously mentioned limitations of the approach, we propose to combine a multilevel approach and a multivariate approach as an interesting alternative. Indeed, the multilevel step splits the different parts of the variation while taking into account the repeated measurements on each subject. Since the stimulation effect from each subject can be separated from the between subject deviation (variation), it is possible to examine the differences in stimulation effect within the subjects in a much easier way than without the separation of the difference sources of variation [19]. Westerhuis et. al (2010) provided the rationale and showed the benefit of the multilevel approach in the analysis of multivariate paired (cross-over) data. In this paper where we aim at identifying genes discriminating the different stimulations, we propose to apply a multivariate approach on the within matrix ***X***_***w***_ which includes the stimulation effect, in the same spirit as in [18-20]. This approach is more powerful, as it takes into account not only the dependency between genes via the multivariate approach, but also the repeated measures between individuals and the stimulation effects via ***X***_***w***_.

#### Extended method for two factors

We propose to extend this approach for data with two factors: the time (‘before’ and ‘after’ vaccination), in addition to the stimulation factor. Let $\left(X_{\mathrm{sjt}}^{k}\right)$ be the expression of a given gene *k* for subject *s* with stimulation *j* at time *t*=1,2. In this context, the mixed model is defined as:

$$\left\{\begin{array}{ll} X_{\mathrm{sjt}}^{k} & =\mu_{\mathrm{jt}}^{k}+\pi_{s}^{k}+{\left(\alpha \pi \right)}_{\mathrm{sj}}^{k}+{\left(\beta \pi \right)}_{\mathrm{st}}^{k}+\varepsilon_{\mathrm{sjt}}^{k},\\ \mu_{\mathrm{jt}}^{k} & =\mu_{\cdot \cdot \cdot }^{k}+\alpha_{j}^{k}+\beta_{t}^{k}+{\left(\alpha \beta \right)}_{\mathrm{jt}}^{k}, \end{array}\right)$$

where for a given gene *k*, $\left(\mu_{\cdot \cdot }^{k}\right)$ is the gene population mean (offset term); $\left(\alpha_{j}^{k}\right)$ measures the fixed effect of stimulation *j*; $\left(\beta_{t}^{k}\right)$ measures the fixed effect of time *t*; $\left({\left(\alpha \beta \right)}_{\mathrm{jt}}^{k}\right)$ is the interaction effect between the stimulation *j* and the time *t*; $\left(\pi_{s}^{k}\overset{}{∼}\;N(0,\sigma_{\pi_{k}}^{2})\right)$ is the random subject effect; $\left({\left(\alpha \pi \right)}_{\mathrm{sj}}^{k}\overset{}{∼}\;N(0,\sigma_{\alpha \pi_{k}}^{2})\right)$ measures the random interaction effect between the subject *s* and the stimulation *j*; $\left({\left(\beta \pi \right)}_{\mathrm{st}}^{k}\overset{}{∼}\;N(0,\sigma_{\beta \pi_{k}}^{2})\right)$ measures the random interaction effect between the subject *s* and the time *t*; the residuals $\left(\varepsilon_{\mathrm{sjt}}\overset{}{∼}\;N(0,\sigma_{\varepsilon_{k}}^{2})\right)$ and the variables $\left(\pi_{s}^{k}\right)$, $\left(\varepsilon_{\mathrm{sjt}}^{k}\right)$, $\left({\left(\alpha \pi \right)}_{\mathrm{sj}}^{k}\right)$ and $\left({\left(\beta \pi \right)}_{\mathrm{st}}^{k}\right)$ are assumed to be independent. In the context of our application, the potential subject interactions effect with the stimulation and the time effect are confounded with the residuals terms since only one observation is available per subject for each level of both time and stimulation factors.

According to the mixed model, we have:

(7) $$\begin{array}{l} x_{\mathrm{sjt}}^{k}=\underset{\text{offset term}}{\underset{︸}{x_{\cdot \cdot \cdot }^{k}}}+\underset{\text{between-subject deviation}}{\underset{︸}{\left(x_{s\cdot \cdot }^{k}-x_{\cdot \cdot \cdot }^{k}\right)}}\\ \;+\underset{\text{within-subject deviation}}{\underset{︸}{\left(x_{\mathrm{sjt}}^{k}-x_{s\cdot \cdot }^{k}\right)}} \end{array}$$

where the within-subject deviation can be further decomposed as:

$$\begin{array}{l} (x_{\mathrm{sjt}}^{k}-x_{s\cdot \cdot }^{k})=\underset{\text{Stimulation effect}}{\underset{︸}{\left(x_{\cdot j\cdot }^{k}-x_{\cdot \cdot \cdot }^{k}\right)}}+\underset{\text{Time effect}}{\underset{︸}{\left(x_{\cdot \cdot t}^{k}-x_{\cdot \cdot \cdot }^{k}\right)}}\\ \;+\underset{\text{interaction effect}}{\underset{︸}{\left(x_{\cdot \mathrm{jt}}^{k}-x_{\cdot j\cdot }^{k}-x_{\cdot \cdot t}^{k}+x_{\cdot \cdot \cdot }^{k}\right)}}\\ \;+\underset{\text{random inter: subject}\times \text{Stimulation}}{\underset{︸}{\left(x_{\mathrm{sj}\cdot }^{k}-x_{\cdot j\cdot }^{k}-x_{s\cdot \cdot }^{k}+x_{\cdot \cdot \cdot }^{k}\right)}}\\ \;+\underset{\text{random inter: subject}\times \text{Time}}{\underset{︸}{\left(x_{s\cdot i}^{k}-x_{\cdot \cdot t}^{k}-x_{s\cdot \cdot }^{k}+x_{\cdot \cdot \cdot }^{k}\right)}}\\ \;+\underset{\text{Residual}}{\underset{︸}{\left(x_{\mathrm{sjt}}^{k}-x_{\cdot \mathrm{jt}}^{k}-x_{\mathrm{sj}\cdot }^{k}-x_{s\cdot t}^{k}+x_{\cdot j\cdot }^{k}+x_{\cdot \cdot t}^{k}+x_{s\cdot \cdot }^{k}-x_{\cdot \cdot \cdot }^{k}\right)}} \end{array}$$

The matrix representation gives:

$$\begin{array}{l} \underset{\text{within-subject deviation}}{\underset{︸}{X_{w}}}\\ =\underset{X_{w^{*}}}{\underset{︸}{X_{\text{Stimulation}}+X_{\text{Time}}+X_{\text{Stimulation}\times \text{Time}}+X_{\text{Residual}}}}\\ \;+\underset{\text{random interaction}}{\underset{︸}{X_{\text{subject}\times \text{Stimulation}}+X_{\text{subject}\times \text{Time}}}} \end{array}$$

Similar to the one-factor decomposition, the multivariate approach will be applied on the within matrix $\left(X_{w^{*}}\right)$, which includes stimulation, time and interaction effects.

### Discriminant analysis of one data set

Once the multilevel approach has been applied to split up the variation in the data, a variant of the multivariate approach PLS Discriminant Analysis (called sparse PLS-DA) is applied on the within matrix ***X***_*w*_ or $\left(X_{w^{*}}\right)$ in order to select discriminative genes between the groups of subjects on a single data set.

#### Sparse PLS-DA

Linear Discriminant Analysis (LDA) and Partial Least Squares Discriminant Analysis (PLS-DA, [15]) are exploratory approaches seeking the optimal linear combinations of variables (genes) which best separate the sample groups. PLS-DA has been found to be a promising alternative to LDA since the latter faces numerical limitations when dealing with too many correlated predictors. Let ***X***(*N* × *p*) be the *within* predictor matrix (to improve readability, the subscript _*w*_ is removed) and ***Y***(*N* × *G*) the response dummy matrix indicating the group of each sample. In PLS-DA, ***X*** is column standardized. The PLS-DA objective function to solve can be written as [15]:

(3) $$\underset{u}{\max}\mathrm{cor}(Y,Xu)\mathrm{var}(Xu),$$

where we denote by ***ξ***=***Xu***the discriminant direction vector, which is a linear combination of the original variables. The vector ***u ***is the associated loading vector indicating the weights of each variable in the linear combination ***ξ***. Once step (3) has been performed and the first weight vector ***u***_1_ has been extracted, both matrices ***Y ***and ***X*** are *deflated* such that the following loading vector ***u***_2_ is orthogonal to the previous one. PLS-DA therefore outputs a set of loading weight vectors ***u***_1_***u***_2_,…,***u***_*H*_ and associated discriminant direction vectors ***ξ***_1_***ξ***_2_,…,***ξ***_*H*_, where *H* is the number of PLS-DA dimensions (or deflations).

The sparse version proposed by Lê Cao et al. [16] uses the Lagrange form of PLS-DA to include a *L*_1_ constraint on ***u*** in order to ensure that some *u*_*j*_ will be estimated as exactly zero (*j*=1,…,*p*). Thus, these corresponding variables will not contribute to the discriminant direction. sPLS-DA therefore allows variable selection for choosing the variables that best discriminate/separate the sample groups.

#### Parameters tuning

Two parameters need to be tuned in sPLS-DA: the number of discriminant vectors *H* and the number of variables to select on each dimension (PLS component). Lê Cao et al. [16] showed that for most cases, the user could effectively set *H*=*G*−1. The number of variables to select is, however, a challenging issue as tuning criteria are often limited by the very small number of samples. In this study, we considered two criteria to guide the choice of this parameter, both of them are applied sequentially, dimension per dimension.

*Tuning criterion 1*. One option is to use cross-validation to choose the optimal number of selected variables in order to avoid selection bias [22]. After this step, the full data are analyzed given this tuned parameter. We propose to estimate the generalization error rate using *k* fold cross-validation. In the case of a very small sample size (<15 subjects), the leave-one-out cross-validation (denoted “loo”) can be used instead. In the specific context of repeated measures and in order to respect the data structure, the training set is composed of the measurements on all experimental units except the measurements on one subject *s* which defines the test set denoted $\left(X_{w,s}^{\mathrm{test}}\right)$. The test set prediction is defined by $\left(Y^{\mathrm{test}}=X_{w,s}^{\mathrm{test}}\beta \right)$, where ***β*** is the regression coefficient matrix from sPLS-DA (see [16] for more details). The process is repeated for each subject and the classification error rate is averaged across all subjects. This process is tested for each number of variables to select (see Additional file 1: Figure S3 and Figure S6), and the “optimal” number of variables is then determined when the lowest error rate is obtained.

*Tuning criterion 2*. In the case where the number of subjects is too small, an ad-hoc alternative approach was used on the whole data set by computing *cor*(***YXu***)*var*(***Xu***) for each deflated matrix and with respect to the number of selected variables. This is similar to that proposed by Waaijenborg et al. [10] and Parkhomenko et al. [9]. The number of variables selected is chosen to maximize the criterion value.

### Integrative analysis of two data sets

Similarly to the PLS-DA analysis, a more general PLS multivariate approach can be applied on the matching within matrices ***X***_*w*_ (or $\left(X_{w^{*}}\right)$) and ***Z***_*w*_ (or $\left(Z_{w^{*}}\right)$). For this analysis however, the aim is to integrate two data sets in a non-supervised manner and select correlated variables from both data sets across the subjects.

#### Sparse PLS

Partial Least Square regression (PLS, [23]) is in fact the ancestor of PLS-DA and is applied in a non supervised context, where ***X***(*N* × *p*) and ***Z***(*N* × *q*) are two continuous *within* matrices of two different types of predictors (*e.g.* gene expression and cytokine secretion). In PLS, both ***X*** and ***Z*** are column standardized. To improve readability, the subscript _*w*_ is removed from both these matrices. PLS relates ***X*** and ***Z*** by a linear multivariate model, while also modelling the structure of ***X*** and ***Z***. PLS is particularly useful for analysing noisy, collinear, even incomplete, high dimensional data, see [24] for a review. PLS performs successive decompositions of ***X***and ***Z*** into new variables (component scores) denoted by (***ξ***_1_,…,***ξ***_*H*_) for the X-scores and (***ω***_1_,…,***ω***_*H*_) for the Z-scores. These scores should be few in number (*H* small), orthogonal to each other within each data set, and estimated as linear combinations of the original variables from ***X*** and ***Z*** with their weights coefficients indicated in the associated loading vectors ***u***_*h*_ and ***v***_*h*_(*h*=1,…,*H*) respectively. In matrix representation, we have ***X***=***ΞC***^*T*^ + ***E***, ***Z***=***ΩD***^*T*^ + ***F***, where ***E*** and ***F*** are the residual matrices, and the column matrices in ***C*** and ***D*** are the coefficients from the local regressions of the score vectors ***ξ***_*h*_(***ω***_*h*_) onto the current deflated matrices defined as $\left(X_{h}=X_{h-1}-\xi_{h}c_{h}^{\prime }\right)$ and $\left(Z_{h}=Z_{h-1}-\omega d_{h}^{\prime }\right)$, where $\left(c_{h}=X_{h-1}^{T}\xi_{h}/\xi_{h}^{\prime }\xi_{h}\right)$ and $\left(d_{h}=Y_{h-1}^{T}\omega_{h}/\omega_{h}^{\prime }\omega_{h}\right)$.

PLS relates both matrices by maximising the covariance between each pair of scores (***ξ***_*h*_,***ω***_*h*_). The PLS objective function is:

(4) $$\arg\underset{∥u_{h}∥=1,∥v_{h}∥=1}{\max}\mathrm{cov}(X_{h}u_{h},Z_{h}v_{h})\;h=1\ldots \mathrm{H.}$$

This PLS form is often referred to as “PLS2 mode A” in the literature [25] where, similar to Canonical Correlation Analysis, the aim is to model a ‘bidirectional’ relationship between the two data sets (to maximise the common information between the two data sets), as opposed to a ‘unidirectional’ relationship when using a regression model. The sparse version, sPLS, enables variable selection from both sets by including *L*_1_ penalizations on both ***u***_*h*_ and ***v***_*h*_ simultaneously in (4), which is solved with a Lagrangian form (see [7,8] for more details about the methodology and the algorithm). The result is a subset of correlated variables from both ***X*** and ***Z*** indicated in the loading vectors (***u***_*h*_***v***_*h*_) for each PLS dimension *h*, and a set of score vectors (***ξ***_*h*_***ω***_*h*_) that are useful for graphical representations.

#### Parameter tuning

As an extension to the tuning criterion 2 from the previous section, and similar to what was proposed by Waaijenborg et al. [10] and Parkhomenko et al. [9], the number of PLS components and number of variables to select in each step can be tuned by computing *cov*(***X***_*h*_***u***_*h*_***Z***_*h*_***v***_*h*_), which is the criterion maximized in sPLS2 mode A for each PLS dimension *h* (see equation (4)). For an optimal number of selected variables from both datasets, one would expect this criterion to achieve also a maximum.

## Results and discussion

We first present the results of a short simulation study to show the importance of using a multilevel approach in comparison to a standard sparse partial least square analysis on the original data. We then apply the proposed multilevel approach on an HIV-vaccination study.

### Simulation study

#### Simulated model

A simulation study based on the following mixed effects model was performed:

$$X_{\mathrm{sj}}^{k}=\mu_{j}^{k}+\pi_{s}^{k}+\varepsilon_{\mathrm{sj}}^{k},\;\;\;s=1,\ldots ,12,\;j=1,\ldots ,4,$$

with $\left(\pi_{s}^{k}\;\;∼\;\;N(0,\sigma_{\pi_{k}}^{2})\right)$, $\left(\varepsilon_{\mathrm{sj}}^{k}\;\;∼\;\;N(0,\sigma_{\varepsilon_{k}}^{2})\right)$, where $\left(\pi_{s}^{k}\right)$ and $\left(\varepsilon_{\mathrm{sj}}^{k}\right)$ are independent. From this model, 10 clusters of 100 genes each were generated (*k*=1,…,1000). For any given pair of genes *k*_1_ and *k*_2_ in the same cluster, a pairwise correlation for $\left(X_{\mathrm{sj}}^{k_{1}}\right)$ and $\left(X_{\mathrm{sj}}^{k_{2}}\right)$ is specified by assuming $\left(\mathrm{cor}(\pi_{s}^{k_{1}},\pi_{s}^{k_{2}})=\rho \right)$ and $\left(\mathrm{cor}(\varepsilon_{\mathrm{sj}}^{k_{1}},\varepsilon_{\mathrm{sj}}^{k_{2}})=\rho \right)$, while genes belonging to different clusters are taken to be uncorrelated. The random variables $\left(\pi_{s}^{k}\right)$ and $\left(\varepsilon_{\mathrm{sj}}^{k}\right)$ from same cluster are generated from the multivariate normal distribution $\left(\left(\pi_{s}^{k_{1}},\ldots ,\pi_{s}^{k_{100}})∼N_{100}(0_{100},\Sigma_{\pi }\right)\right)$ where the variance-covariance matrix *Σ*_*π*_ is a (100× 100) matrix with $\left(\sigma_{\pi_{k}}^{2}\right)$ along the diagonal and $\left(\rho \sigma_{\pi_{k}}^{2}\right)$ for the others terms; and from the multivariate normal distribution $\left(\left(\varepsilon_{\mathrm{sj}}^{k_{1}},\ldots ,\varepsilon_{\mathrm{sj}}^{k_{100}})∼N_{100}(0_{100},\Sigma_{\varepsilon }\right)\right)$ where the variance-covariance matrix *Σ*_*ε*_ is a (100×100) matrix with $\left(\sigma_{\varepsilon }^{2}\right)$ along the diagonal and $\left(\rho \sigma_{\varepsilon_{k}}^{2}\right)$ for the others terms.

To mimic the application, clusters of genes discriminating 4 conditions were generated (the 4 stimulations denoted LIPO5, GAG+, GAG- and NS) , where the mean effect of each stimulation is specified by $\left(\mu^{k}={\left(\mu_{1}^{k},\mu_{2}^{k},\mu_{3}^{k},\mu_{4}^{k}\right)}^{T}\right)$, according to the following: 

• 2 gene clusters discriminate (LIPO5, GAG+) versus (GAG-, NS) with ***μ***^*k*^=(4,4,0,0)^*T*^and ***μ***^*k*^=(3,3,0,0)^*T*^.

• 2 gene clusters discriminate LIPO5 versus GAG+, while GAG+ and NS have the same effect: ***μ***^*k*^=(5,2,0.2,0.2)^*T*^and ***μ***^*k*^=(5,2,0,0)^*T*^.

• 2 gene clusters discriminate GAG- versus NS, while LIPO5 and GAG+ have the same effect: ***μ***^*k*^=*c*(1,1,5,2)^*T*^and ***μ***^*k*^=*c*(0,0,5,2)^*T*^.

• the 4 remaining clusters represent noisy signal (no stimulation effect): ***μ***^*k*^=*c*(0,0,0,0)^*T*^and ***μ***^*k*^=(0.5,0.5,0.5,0.5)^*T*^.

The intra cluster correlation was either set to *ρ*=0.7 or 0.8. Different values for $\left(\sigma_{\pi_{k}}^{2}\right)$ and $\left(\sigma_{\varepsilon_{k}}^{2}\right)$ were studied, but for the sake of conciseness the results are only presented for $\left(\sigma_{\pi_{k}}=2\right)$ and $\left(\sigma_{\varepsilon_{k}}=0.5\right)$.

### Numerical results

**Figure 1:**
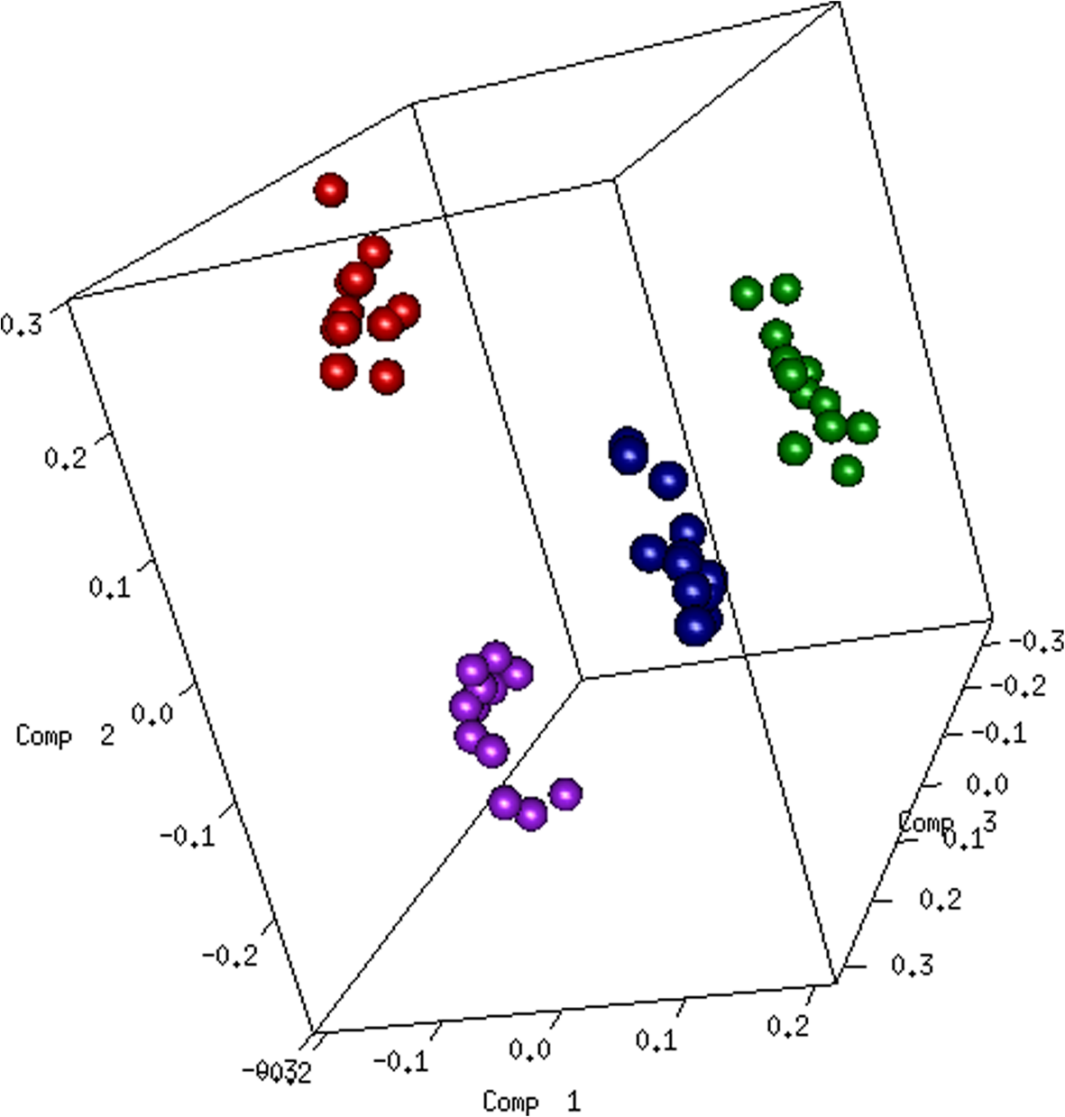
**Simulation study.** Sample representation from multilevel sPLS-DA. Samples were projected onto a subspace spanned by the first 3 sPLS-DA components, based on the 200 genes selected on each of the 3 components.

From the simulated data, the within matrix was computed and applied to multilevel sPLS-DA. Figure 1 displays the sample representation for the first 3 axes or dimensions for one simulation run.

**Figure 2:**
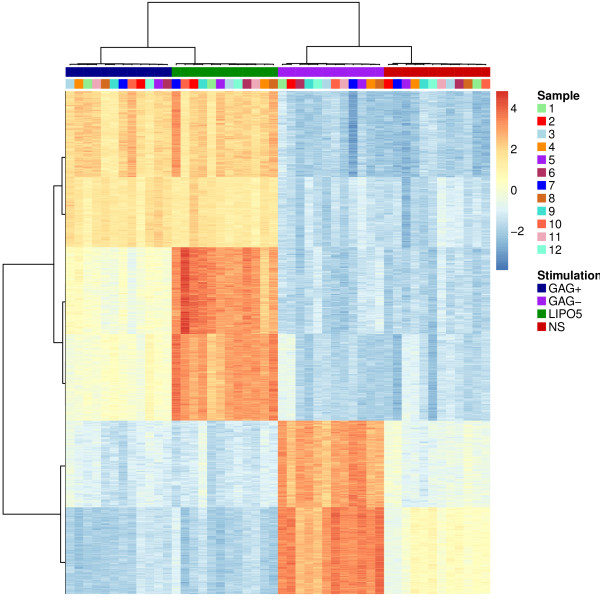
**Stimulation study.** Hierarchical clustering (Euclidian distance and Ward method aggregation) of the genes selected with multilevel sPLS-DA. Samples are represented in columns and genes in rows.

Firstly, in order to highlight the benefit of the multilevel approach in comparison to the multivariate approach without the split-up variation step, a prespecified number of genes was selected on each dimension in order to assess the ability of each approach to select the true relevant genes. As expected, 3 components (linear combinations of 200 genes) were sufficient to discriminate the effect of the 4 stimulations. Multilevel sPLS-DA (applied on the within matrix) selected 92% of the true simulated discriminative genes as compared to 75% of the true discriminative genes for classical sPLS-DA (applied on the original matrix), see Table 1. The hierarchical clustering of the genes selected by sPLS-DA on the within matrix (Figure 2) confirmed the discriminatory ability of these genes to separate the 4 groups of samples. As expected, a group of 6 gene clusters can be observed. On the contrary, we did not observe such clusters when applying sPLS-DA on the original matrix (not shown).

**Table 1:** Simulation study

	**Component 1**	**Component 2**	**Component 3**	**All**
classical sPLS-DA	58.0	75.0	87.2	78.2
multilevel sPLS-DA	82.8	95.6	93.1	92.0

Percentage of the number of true selected genes selected by classical sPLS-DA or multilevel sPLS-DA on each component or dimension (averaged over 100 simulation runs); 200 genes were selected on each component.

Secondly, leave-one-out cross-validation was performed on each simulation run to evaluate the error rate of classification of classical sPLS-DA or multilevel sPLS-DA (Table 2). The classification error rate was evaluated for different number of genes selected on each component. For example, the average classification error rate for multilevel sPLS-DA was of 0.009 for 200 genes selected on each of the 3 axes compared to an error rate of 0.268 for the same parameters with classical sPLS-DA.

**Table 2:** Simulation study

**Number of genes**	**Original matrix**			**Within matrix**		
	**1 component**	**2 components**	**3 components**	**1 component**	**2 components**	**3 components**
25	0.535	0.369	0.312	0.500	0.271	0.024
50	0.530	0.364	0.311	0.500	0.265	0.016
75	0.527	0.360	0.306	0.500	0.261	0.013
100	0.524	0.354	0.300	0.500	0.258	0.011
125	0.522	0.351	0.296	0.500	0.257	0.009
150	0.520	0.343	0.285	0.500	0.250	0.008
175	0.518	0.335	0.281	0.500	0.243	0.009
200	0.516	0.327	**0.268**	0.500	0.234	**0.009**
225	0.514	0.323	0.269	0.500	0.227	0.009
250	0.512	0.316	0.267	0.500	0.220	0.008
275	0.510	0.314	0.266	0.500	0.207	0.007
300	0.510	0.306	0.262	0.500	0.196	0.007
325	0.509	0.299	0.260	0.500	0.182	0.007

Classification error rate estimation using leave-one-out cross-validation for classical sPLS-DA and multilevel sPLS-DA, with respect to the number of genes selected on each component (averaged over 100 simulation runs).

### Application to HIV vaccine evaluation

#### Description of the study

The data come from a trial evaluating a vaccine based on HIV-1 lipopeptides in HIV-negative volunteers [26]. The vaccine (HIV-1 LIPO-5 ANRS vaccine) contains five HIV-1 amino acid sequences coding for Gag, Pol and Nef proteins. A subsample of 12 vaccinated participants was randomly selected and experiments were performed before and after vaccination. The data consist of monitored cytokine secretion and gene expression measurements from purified *in vitro* stimulated Peripheral Blood Mononuclear Cells (PBMC). Cytokine secretion was analysed by cytokine multiplex (millipore) in the supernatant of PBMC after 11-day-culture, and the data set consists of 10 cytokines measurements (IFN*γ*, IL1*β*, IL2, IL5, IL6, IL10, IL13, IL17, IL21, and TNF*α*). Gene expression was analysed using the Illumina HumanHT-12 v4 Expression BeadChip on PBMC before (W0) and 14 weeks after vaccination (W14), 6 hours after *in vitro* stimulation by either (1) all the peptides included in the vaccine (LIPO-5), or (2) the Gag peptides included in the vaccine (GAG+) or (3) the Gag peptides not included in the vaccine (GAG-) or (4) without any stimulation (NS).

### Preprocessing

Background correction, log_2_ transformation and quantile normalisation were applied on the gene expression data using the **R**limma package. Probes were further prefiltered for each time point (before and after vaccination) using a P-value detection (<1% in all samples). The preprocessed data set contained the expression of 25,109 probes for 12 subjects for 4 types of stimulation before vaccination (W0) and the expression of 24,687 probes after vaccination (W14). Some samples were not available due to DNA quality issues, resulting in 44 samples at W0 and 42 samples at W14. For the multilevel approach with two factors, the analysis was performed on the common prefiltered probes before and after vaccination (21,350 probes in total).

The statistical analysis was performed on the probe expression, but the results were biologically interpreted at the gene level.

#### Discriminant analysis on the transcriptomics data

First we present results obtained using a mixed model and discuss some potential limitations of this method in the context of small sample size. Then we present the results obtained using multilevel sPLS-DA for one and two-factor analyses. To shorten the length of the paper, some results have been moved in Additional file 1. The R code used for the analysis of this study is provided in Additional file 2.

### Mixed model

The one-level mixed model was applied to the W14 transcriptomics data. We used the mle function from the R package nlme with the maximum likelihood method for the estimation of the different models. A global test (likelihood ratio test) followed by an FDR multiple correction (5%) identified 2308 DE genes in at least one of the stimulation. Pairwise comparisons based on Wald test (FDR = 5%) were then performed to compare LIPO5 vs. NS (2108 DE genes), GAG+ vs. NS (1087 DE genes) and GAG-vs. NS (209 DE genes). The summary of the results is available in Section 1 of the Additional file 1. In our case study, the clustering analysis of the 100 most significant differentially expressed genes selected by the mixed model failed to discriminate the four stimulations (Additional file 1: Figure S2).

The univariate mixed model approach is commonly used to analyse data with repeated measurement with an unbalanced design. However, several reasons favor the use of a multilevel approach in this high dimensional setting. Apart from the already mentioned problem of numerous independent tests and the requirement to apply multiple correction [27], another limitation is the sensitivity of the FDR threshold (and therefore the number of declared DE genes) to the total number of test performed. The latter depends on the preprocessing method used to filter the probes. Another issue encountered was problems of convergence with both the maximum likelihood (ML) and the restricted ML methods due to the small number of samples in this data set. The asymptotic likelihood ratio test used for fixed effects has been reported to be anti-conservative in [28]. The authors recommended to use the F-test which still poses the issue of the choice of the number of degrees of freedom with a small number of samples [29,30].

### Multilevel approach with one factor

A multilevel sPLS-DA analysis was performed on the W14 transcriptomics data, with *H*=3. Respectively 30, 137 and 123 genes were selected with the approach on each dimension according to the tuning criterion 1 for the most parcimonious model. Although *k* fold cross-validation would have been preferable to use, loo was used in this study given the small number of subjects. The following ‘loo’ classification error rates (0.48, 0.26, 0.24) were obtained on the first three sPLS-DA dimensions compared to (0.48, 0.36, 0.38) when applying sPLS-DA on the original matrices (see Additional file 1: Figure S3).

**Figure 3:**
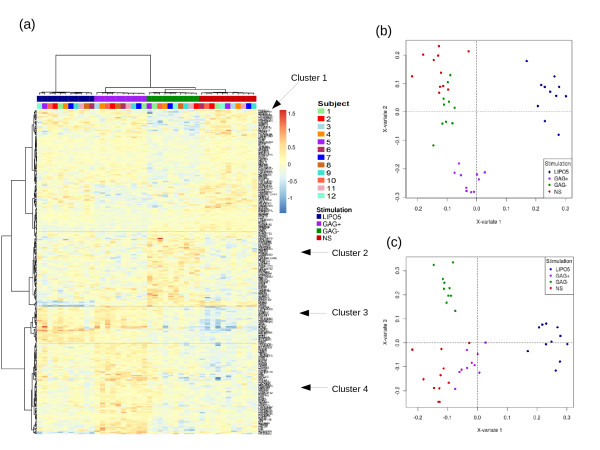
**Multilevel sPLS-DA analysis on the transcriptomics data with one factor (W14).(a)** Unsupervised clustering analysis with Euclidian distance and Ward method of the 290 genes selected by sPLS-DA. Samples are represented in columns and genes in rows. **(b)** and **(c)** sPLS-DA sample representation for dimensions 1-2 **(b)** or 1-3 **(c)**.

Given the expression of these 290 selected genes, Figures 3(b) and 3(c) highlight a good separation between the four stimulations. These sample representations obtained from sPLS-DA reveal that the first component discriminates the stimulation LIPO5 versus the other stimulations, while the second component discriminates the stimulation GAG+ versus the other stimulations and the third component discriminates the stimulation GAG- versus the others. Therefore, the first two components separated the stimulations according to the peptides included in the vaccine. As expected there was a clear separation between LIPO5 and other stimulation conditions. Especially, a part of the differential effect of LIPO5 compared to GAG+ could be due to the lipid tail or perhaps to the effect of the other peptides of LIPO5. GAG- and NS were not distinguishable on the first two components of the sPLS-DA (Figure 3(b)). This last result is not surprising as no specific response is expected from peptides not included in the vaccine after vaccination.

Figure 3(a) displays the unsupervised clustering of the 290 selected probes. A cluster of 11 genes MT1M, C20ORF127, MT2A, MT1A, MT1G, MT1F, LOC441019, MT1X, MT1H, MTE, MT1E, was removed to improve visualisation. These genes selected on dimension 1 were all overexpressed in LIPO5 stimulation (see Additional file 1: Figure S5).

Several clusters of genes which expression seemed related to each type of stimulation could be identified. Cluster 1 included a subset of genes downregulated in GAG-, in cluster 2 the genes were overexpressed in GAG-, while cluster 3 included a subset of genes overexpressed in LIPO5 and GAG+, and cluster 4 was composed of a subset of genes mainly overexpressed in GAG+. The advantage of sPLS-DA is its ability to select genes related to a specific stimulation group on each component. For instance, clusters 1 and 2 included 126 out of the 137 probes selected on the third dimension which separated GAG- from the other stimulation groups (Figure 3(c)). Cluster 3 included 19 out of the 30 probes selected on the first component, and 12 probes from the second component in order to discriminate stimulations LIPO5 and GAG+, while the fourth cluster included 72 out of the 123 probes selected on the second component which separated GAG+ vs. the other stimulations (Figure 3(b)). Interestingly, some of the genes in this cluster belong to the TNF family (TNFSF13B) or interferon family (ISG20L2) demonstrating a specific effect of the GAG peptides on gene expression related to the immune response.

Note that the same analysis was also performed on W0 but identified much fewer discriminative genes (30 genes in total), indicating that there was a change in expression level after vaccination (see Additional file 1: Figure S8).

### Multilevel approach with two factors

A multilevel sPLS-DA analysis was performed on the within matrix $\left(X_{w^{*}}\right)$ including the time factor W0 and W14 in addition to the stimulation factor for the transcriptomics data. The complexity of this cross-over design implied more conditions (4× 2=8) to be compared for 12 unique subjects. Therefore, the tuning criterion 2 gave for each sPLSDA dimension a maximum correlation of (0.94, 0.96, 0.95) for variable selection sizes of 30, 40, 150 genes on each dimension. A sudden drop in the correlation value in the fourth dimension (0.62) indicated that 3 sPLS-DA dimensions should be chosen for this analysis.

**Figure 4:**
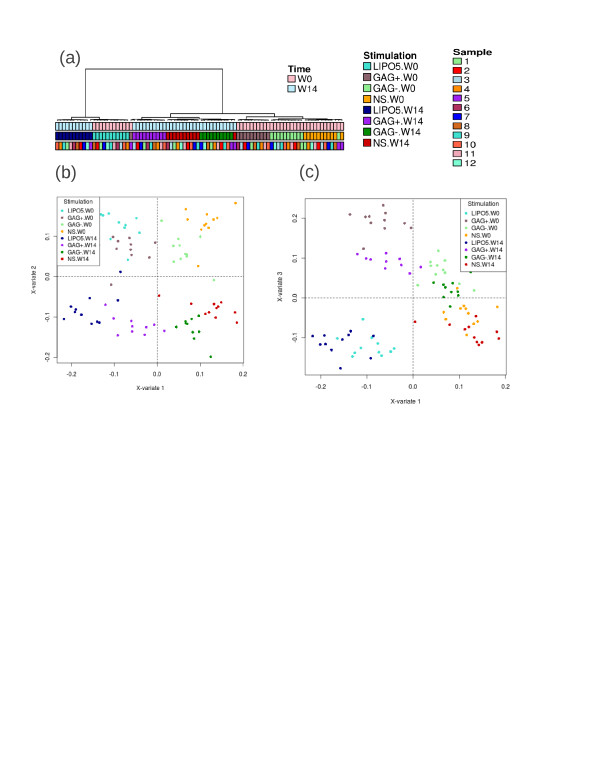
**Multilevel sPLS-DA analysis on the transcriptomics data with two factors stimulation and time.(a)** Unsupervised clustering analysis with Euclidian distance and Ward method of the 220 genes selected by sPLS-DA. sPLS-DA sample representations for dimensions 1-2 **(b)** or 1-3 **(c)**.

The hierarchical clustering of the 220 selected genes indicated a very satisfying separation of both time and stimulation factors (Figure 4(a)). The first component discriminated the stimulations GAG+/LIPO5 vs. GAG-/NS irrespective of the time, whereas the second component reflected the time effect W0 vs. W14 (Figure 4(b)). This suggests that the stimulation groups are easier to separate than the time points by the approach. On this second dimension, relevant genes related to the immune response were selected (CD8a, CD79a, CD19, SLAMF6). The third component (Figure 4(c)) separated GAG+ vs. LIPO5 irrespective of the time and several of the genes selected on this third dimension were found to be metallothionein genes (MT1M, MT2A, MT1A, MT1G, MT1F, MT1X, MT1H, MTE, MT1E) that may be stimulated by the lipid tail of LIPO5. From a biological point of view, the significance of genes from metallothionein family in the context of HIV is not clear although some results have been recently reported [31]. These authors showed an increased resistance to apoptosis of immune-activated monocyte linked to the increase in Metallothionein (MT) gene expression and intracellular zinc levels.

#### Integrative analysis

Multilevel sPLS enables the integration of data measured using different assays. This approach differs from multilevel sPLS-DA as the aim is to select subsets of genes and cytokines which are highly correlated (positively or negatively) across the samples. While the paired structure of the data is still taken into account in the analysis via the decomposition of the within matrices $\left(X_{w}^{*}\right)$ and $\left(Z_{w}^{*}\right)$, the analysis is completely unsupervised: no prior knowledge about the samples groups is included.

### Multilevel approach

Multilevel sPLS was applied on the within matrices of the gene and cytokine data sets after vaccination. Given the very small number of cytokines, all cytokines were selected in the model, and the tuning of the number of variables to select was only performed on the gene expression data set. Respectively, a selection of 50, 1 and 60 genes was performed each of the sPLS dimension, corresponding to a correlation of (0.86, 0.62 and 0.84). A drop of the subsequent correlations for the other dimensions guided the choice of 3 components in the model.

**Figure 5:**
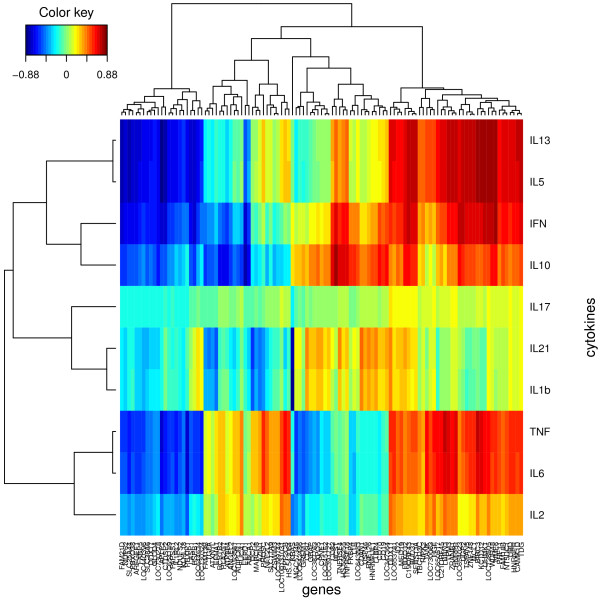
**Integrative analysis of gene expression and cytokine secretion for W14.** Clustered Image Maps (CIM) obtained from multilevel sPLS. Selected genes are represented in columns and cytokines in rows.

Although unexpected and indicated by the tuned correlation value of 0.62, the selection of one single gene on the second dimension was not surprising given the sample representation that was obtained (see Additional file 1: Figure S11): while the first and third dimensions separated LIPO5, GAG+ and GAG-/NS, the second dimension did not seem to highlight any interesting pattern in the data. The approach might reveal some unknown phenomenon in the data for this component that would need to be further investigated. Nonetheless, sPLS multilevel was able to identify very relevant information from both data sets. Graphical tools help to unravel the correlation structure between the two data set such as Clustered Image Maps (CIM). Figure 5 reveals clusters of selected genes associated with cytokines secretion. These genes were not known to participate in the cytokines pathways but can be seen as gene signatures to predict future cytokine response. For example Figure 5 highlights relevant clusters of cytokines, such as the proximity of the two T-Helper type 2 (Th2) cytokines IL5 and IL13. Also, IL17 and IL21 have often been associated in the type 17 response. The correlations between genes and cytokines were similar for the pairs (IL5, IL13), (IL21,IL1b) and (TNF,IL6) underlying potential similar pathways related to the production of these cytokines.

## Conclusion

In this paper, we have proposed a two-step analysis combining a multilevel approach and a multivariate approach to analyze repeated measures of gene expression. The multilevel approach first extracts the within-sample variation while the multivariate approach applied on the within matrix takes into account the dependency between the variables. The multilevel approach was extended for one and two factors analyses.

Two multilevel variants were proposed with either sPLS-DA or sPLS. The multilevel sPLS-DA approach selects genes separating the groups of subjects on a single data set. The simulation study comparing multilevel sPLS-DA and the sPLS-DA applied on the original data demonstrated the good performance of the model. The multilevel sPLS approach integrates two experiments made on different platforms but on the same subjects, and selects subsets of correlated variables from both sets.

The application of both types of approaches on the HIV-1 vaccine trial showed their ability to highlight the stimulation groups and to select biologically relevant genes related to immune response. Hence, our combined multilevel approach may help in finding signatures of vaccine effect and allows for a better understanding of immunological mechanisms activated by the intervention. Future work will include a thorough analysis on the gene/probe annotations to fully understand the mechanistic link between gene differential expression, cytokine secretion according to the various stimulations.

## Endnote

^a^http://www.math.univ-toulouse.fr/~biostat/mixOmics.

## Competing interests

The authors declare that they have no competing interests.

## Authors’ contributions

BL and KL developed the methodology, the R code, performed the simulation and the analysis on the dataset as well as wrote the manuscript. RT developed the methodology, interpreted the dataset as well wrote the Manuscript. HH collected the data and wrote the Application section. All authors read and approved the final manuscript.

## Supplementary Material

Additional file 1 Supplementaries results regarding the VAC18 study experiments from two assays. Click here for file

Additional file 2 R code used for the analysis of the VAC18 study. Click here for file
