# Supplementary material for: A novel approach for biomarker selection and the integration of repeated measures experiments from two assays
Source: BMC Bioinformatics. 2012 Dec 6;13:325. doi: 10.1186/1471-2105-13-325 (PMC3627901; doi:10.1186/1471-2105-13-325)
Supplement: Additional file 1 — Supplementaries results regarding the VAC18 study experiments from two assays. [file 1471-2105-13-325-S1.pdf]

Supplementary material: A novel approach for biomarker selection and the integration of repeated measures experiments from two assays

August 9, 2012

Liquet Benoît [1,2], Lê Cao Kim-Anh [3], Hakim Hocini [4,5], and Thiébaut Rodolphe [2,5]

[1]Univ. Bordeaux, ISPED, centre INSERM U-897-Epidémiologie-Biostatistique, Bordeaux, F-33000, FRANCE
[2]INSERM, ISPED, centre INSERM U-897-Epidémiologie-Biostatistique, Bordeaux, F-33000, FRANCE
[3]Queensland Facility for Advanced Bioinformatics, The University of Queensland, QLD 4072, Australia
[4]INSERM U955 Eq 16, UPEC Université, Créteil
[5]Vaccine Research Institute ANRS, France

1 Discriminant Analysis of one data set

1.1 Analysis with one factor

1.1.1 Mixed model approach

The main results obtained with the mixed model (transcriptomics data after vaccination) are represented by the Venn diagram in Figure 1. Figure 2 represents a heatmap of the 100 most significant differentially expressed (DE) genes selected by the mixed model amongst a total of 2308 DE genes (2108 LIPO5 versus NS; 1087 GAG versus NS; 187 GAG- versus NS).

1.1.2 Multilevel sPLS-DA

After vaccination. The classification error rate of multilevel sPLS-DA (transcriptomics data) was estimated with leave-one-out cross validation with respect to the number of selected genes on each sPLS-DA component or dimension. The number of genes to select was tuned sequentially, one dimension at a time and led to an optimal selection of 30, 137 and 123 genes on each dimension (Figure 3) corresponding to error rates equal to (0.48, 0.26, 0.24) according to 'loo' compared to (0.48, 0.36, 0.38) when applying sPLS-DA on the original matrices. Given the expression of these 290 selected genes by multilevel sPLS-DA, Figure 4 highlights a good separation between the four stimulations.

Figure 5 represents the hierarchical clustering of the 290 genes selected by the multilevel sPLS-DA. A cluster of genes highly over expressed in stimulation LIPO5 (MT1M, C20ORF127, MT2A, MT1A, MT1G, MT1F, LOC441019, MT1X, MT1H, MTE, MT1E) was removed for better interpretability of the other genes.

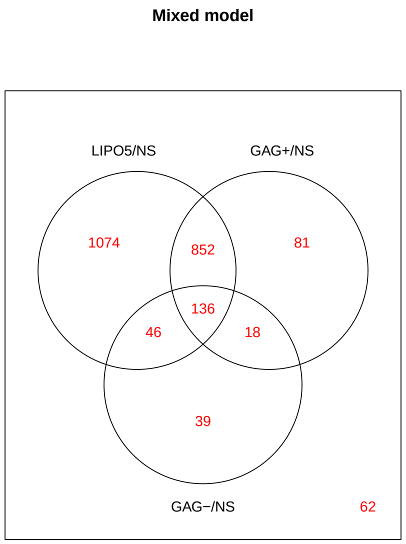

Figure 1: HIV study (transcriptomics data after vaccination). Venn diagram for the differentially expressed genes using the mixed model approach (3 pairwise comparisons).

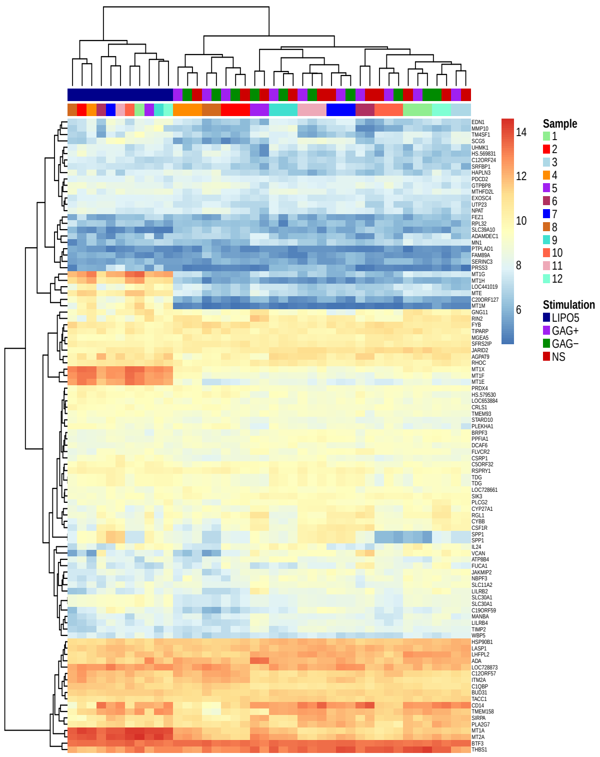

Figure 2: HIV study (transcriptomics data after vaccination). Hierarchical clustering (Euclidian distance and "ward" aggregation) of the 100 most differentially expressed genes (out of 2308) identified by the mixed model (after vaccination). Samples are represented in columns and genes in rows.

Before vaccination. The classification error rate of multilevel sPLS-DA (transcriptomics data) was estimated with leave-one-out cross validation with respect to the number of selected genes on each sPLS-

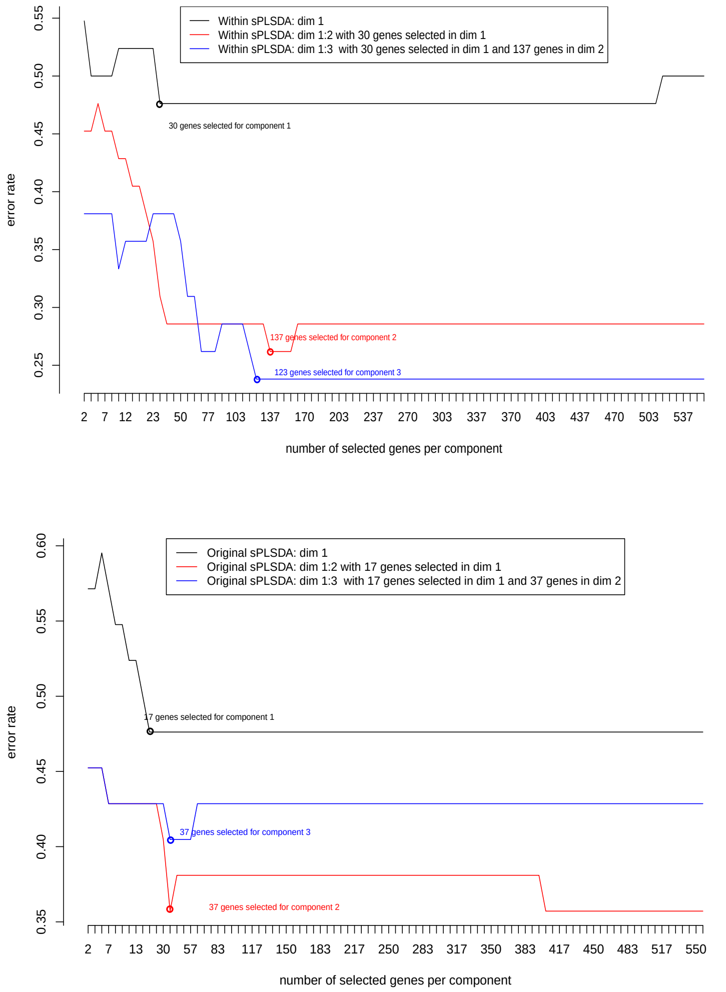

Figure 3: HIV study (transcriptomics data after vaccination). Classification error rate (leave-one-out cross-validation performed sequentially on each dimension) for multilevel sPLS-DA (top) and classical sPLS (bottom). The optimal number of genes selected on each dimension is indicated on the plot.

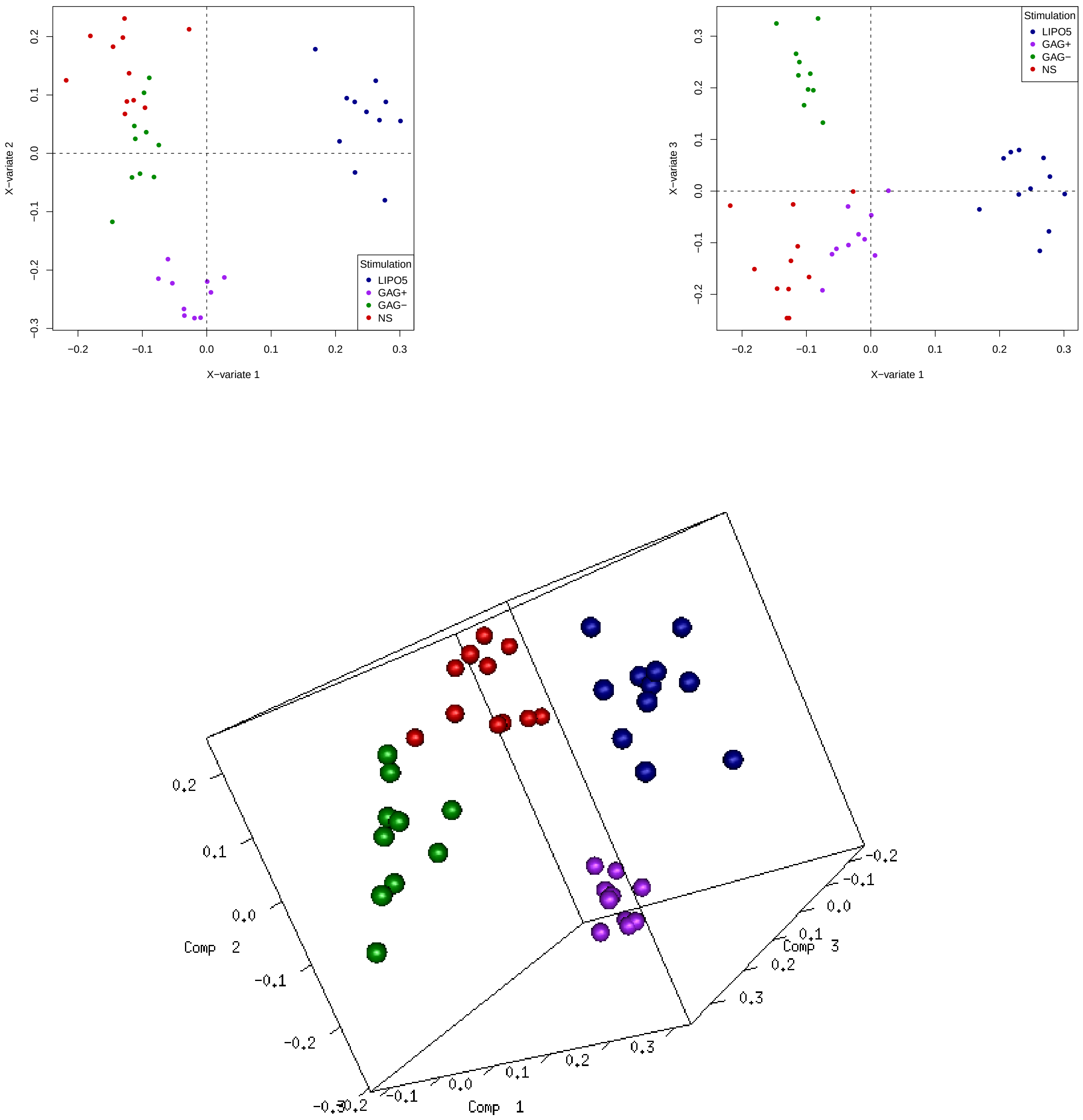

Figure 4: HIV study (transcriptomics data after vaccination). Sample representation with multilevel sPLS-DA for dimensions 1-2 (top left), 1-3 (top right) or with all 3 dimensions (bottom).

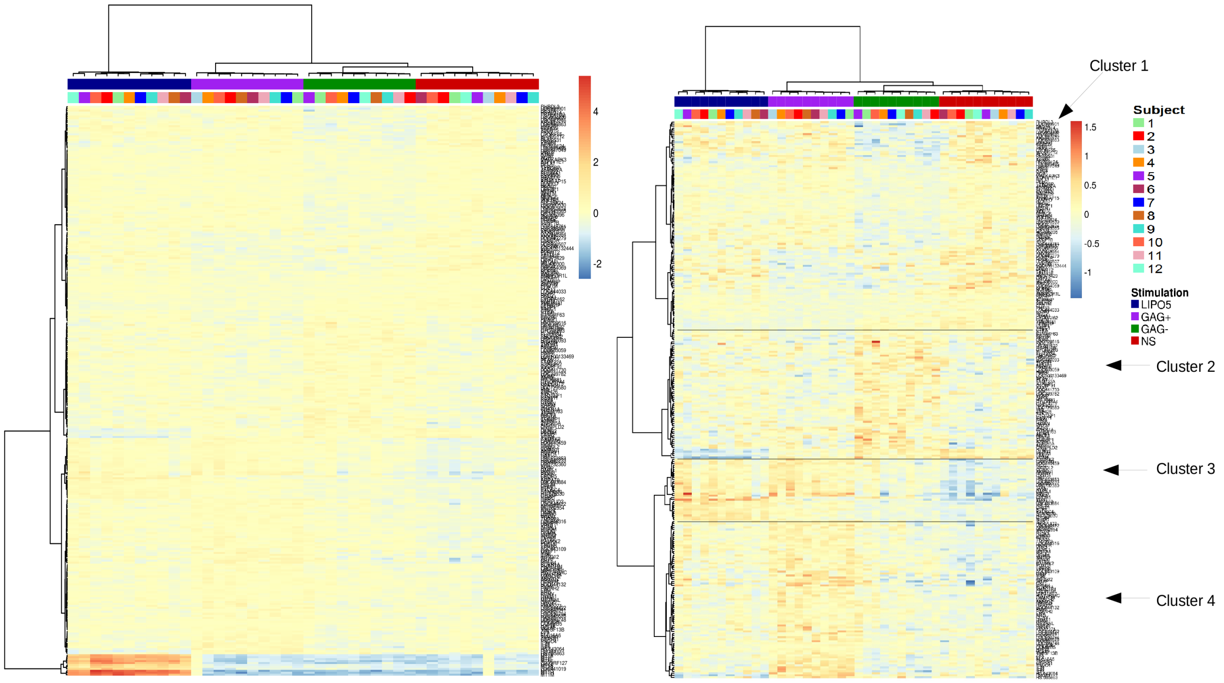

Figure 5: HIV study (transcriptomics data after vaccination). Hierarchical clustering (Euclidian distance and ”ward” method aggregation) of the 290 genes selected with multilevel sPLS-DA (left). One cluster of genes over-expressed in LIPO5 has been removed (right). Samples are represented in columns and genes in rows.

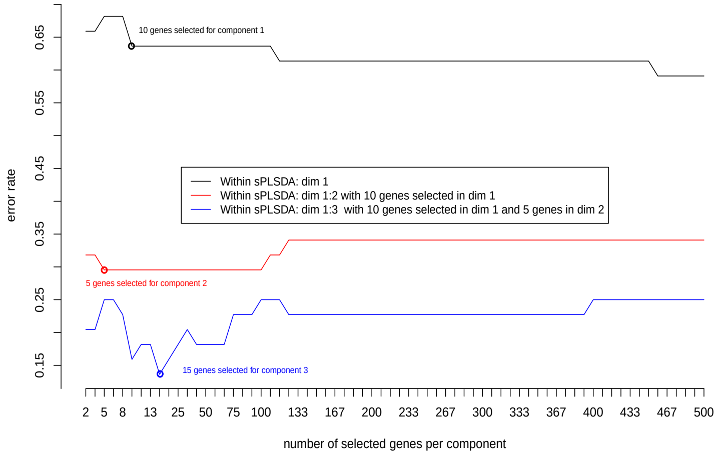

Figure 6: HIV study (transcriptomics data before vaccination). Classification error rate (leave-one-out cross-validation performed sequentially on each dimension) for multilevel sPLS-DA

DA component or dimension. The number of genes to select was tuned sequentially, one dimension at a time and led to an optimal selection of 10, 5 and 15 genes on each dimension (Figure 6). Given the expression of these 30 selected genes by multilevel sPLS-DA, Figure 7 highlights a good separation between the four stimulations. Figure 8 represents the hierarchical clustering of the 30 genes selected by the multilevel sPLS-DA.

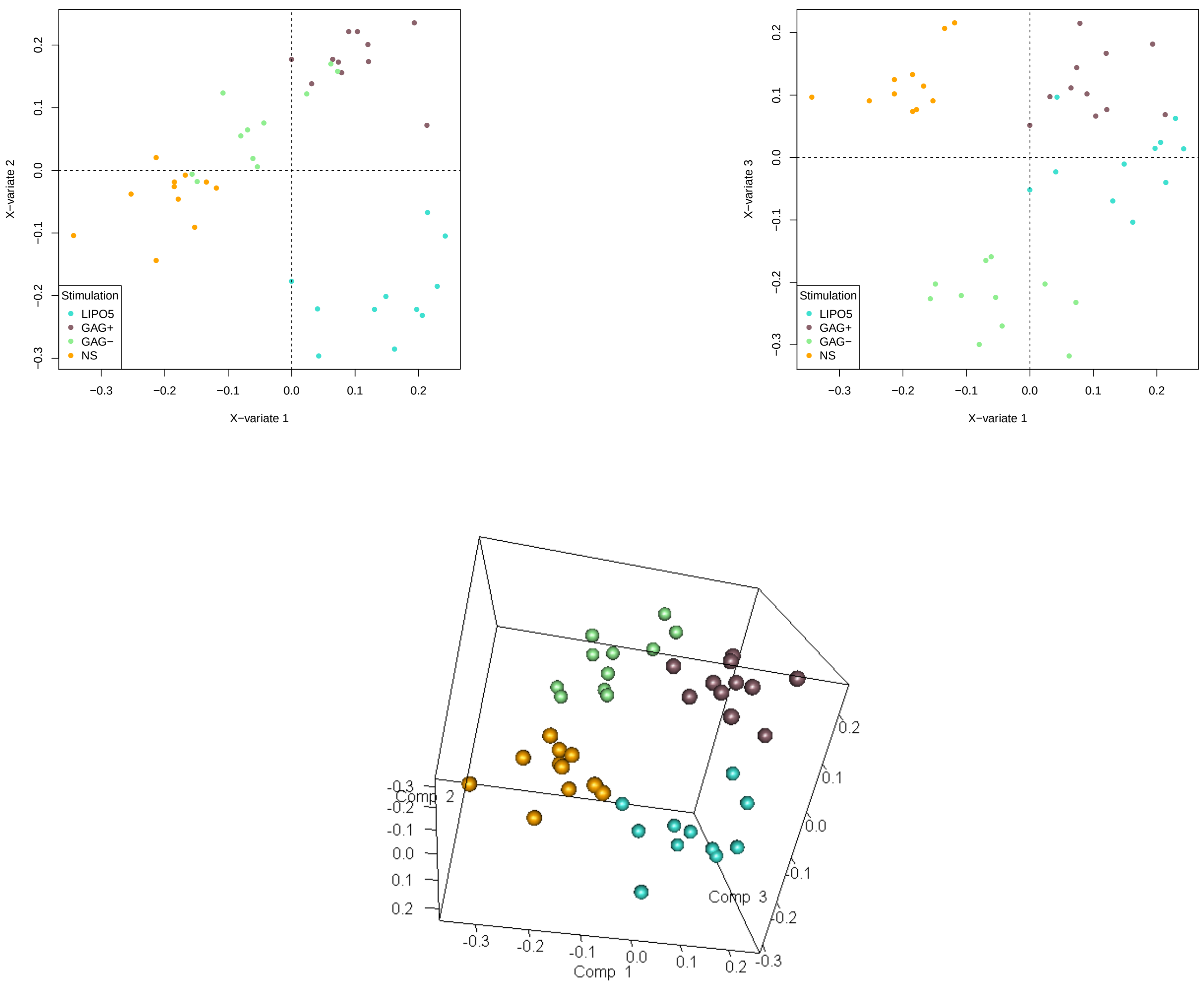

Figure 7: HIV study (transcriptomics data before vaccination). Sample representation with multilevel sPLS-DA for dimensions 1-2 (top left), 1-3 (top right) or with all 3 dimensions (bottom).

1.2 Analysis with two factors

Figure 9 displays the sample representation with multilevel sPLS-DA on the transcriptomics data for the 4 stimulations before and after vaccination. A selection of 30, 40 and 150 genes were chosen on each of the sPLS-DA dimensions. Figure 10 displays the unsupervised clustering of the 220 selected probes.

2 Integrative Analysis

Sample representation of the integrative analysis with sPLS multilevel illustrates how the combinations of components 1 and 3 are able to separate three groups: LIPO5, GAG+ and GAG-/NS (see Figure 11).

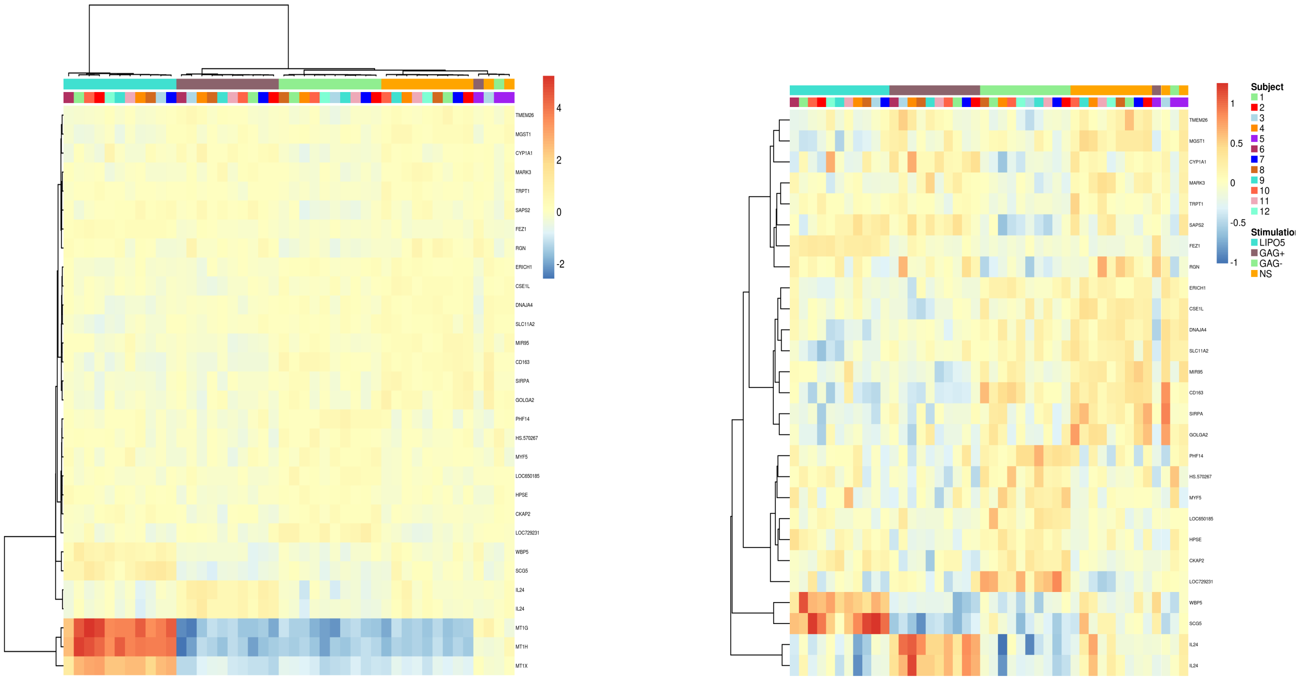

Figure 8: HIV study (transcriptomics data before vaccination). Hierarchical clustering (Euclidian distance and "ward" method aggregation) of the 30 genes selected with multilevel sPLS-DA (left). One cluster of genes over-expressed in LIPO5 has been removed (right). Samples are represented in columns and genes in rows.

According to Tenenhaus (1998), the part of variance of X explained by its own component t_1 is defined as:

$$Rd(X;t_1) = \frac{1}{p}\sum_{j=1}^{p} cor^2(x_j, t_1)$$

Similarly, once can compute the part of variance of X explain by the component of the other data set u_h. And the part of variance explained by all components is defined as:

$$Rd(X;t_1,t_2,...,t_H) = \frac{1}{p}\sum_{j=1}^{p} cor^2(x_j,t_1,t_2,...,t_H) = \frac{1}{p}\sum_{h=1}^{H}\sum_{j=1}^{p} cor^2(x_j,t_1,t_2,...,t_h) = \sum_{h=1}^{H} Rd(X;t_h)$$

	RdX.t	RdX.u	RdZ.t	RdZ.u
dim1	0.08	0.06	0.34	0.44
dim2	0.05	0.05	0.11	0.18
dim3	0.05	0.05	0.15	0.16
sum	0.18	0.15	0.60	0.78

Table 1: Part of explained variance of X_w and Z_w with respect to the sPLS components for each dimension.

Table 1 gives the part of explained variance for each data set and each type of sPLS components. The sPLS components associated to their own data set (t_h for X_w, and u_h for Z_w) explain a bigger part of the

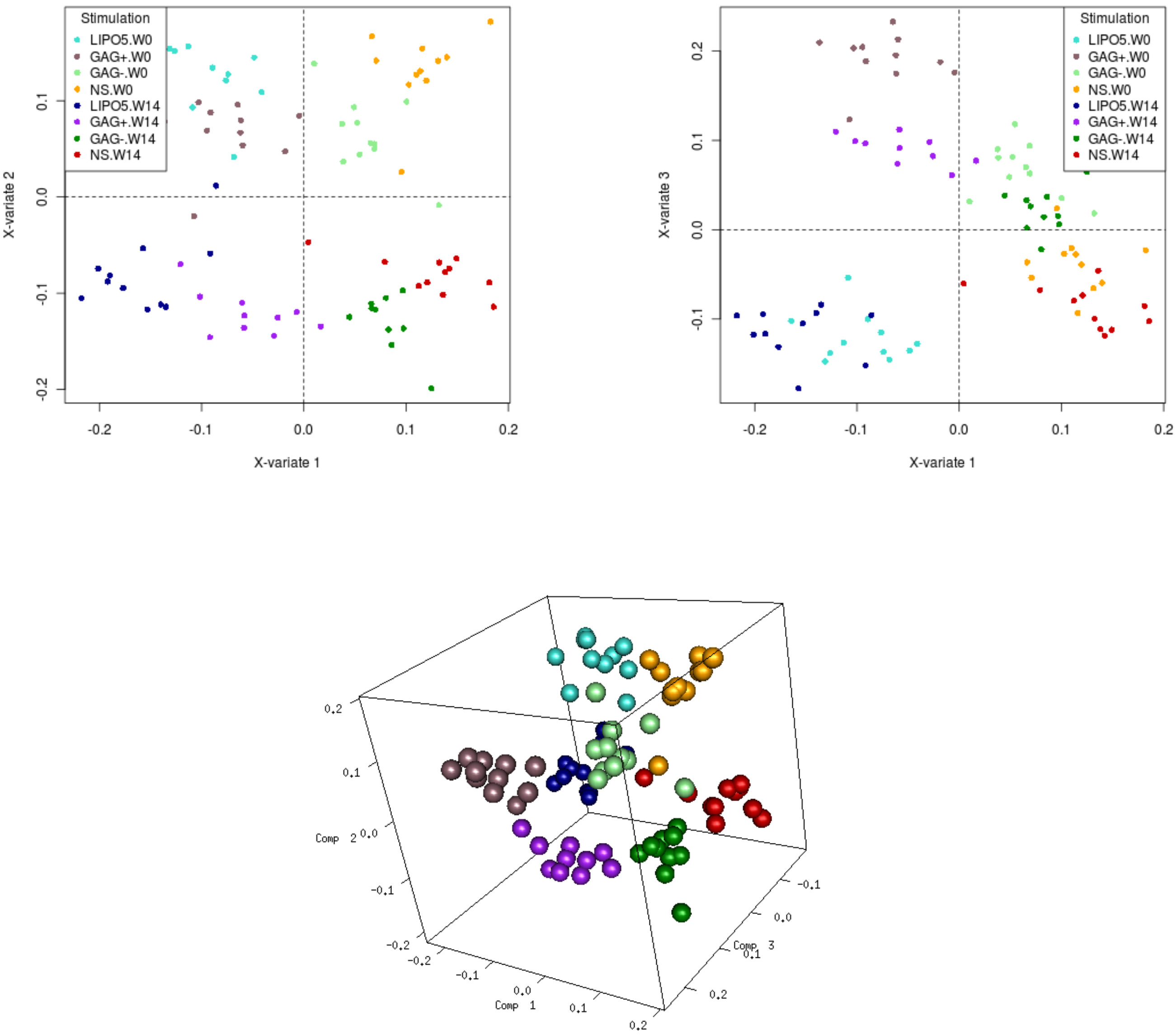

Figure 9: HIV study (transcriptomics data before and after vaccination). Sample representation with multilevel sPLS-DA for dimensions 1-2 (top left), 1-3 (top right) or with all 3 dimensions (bottom).

variance than to the other data set, which is to be expected in this modelling. As often observed in high dimensional data sets, the part of the variance in X_w is very low (18% of the total variance in X_w for 3 components).

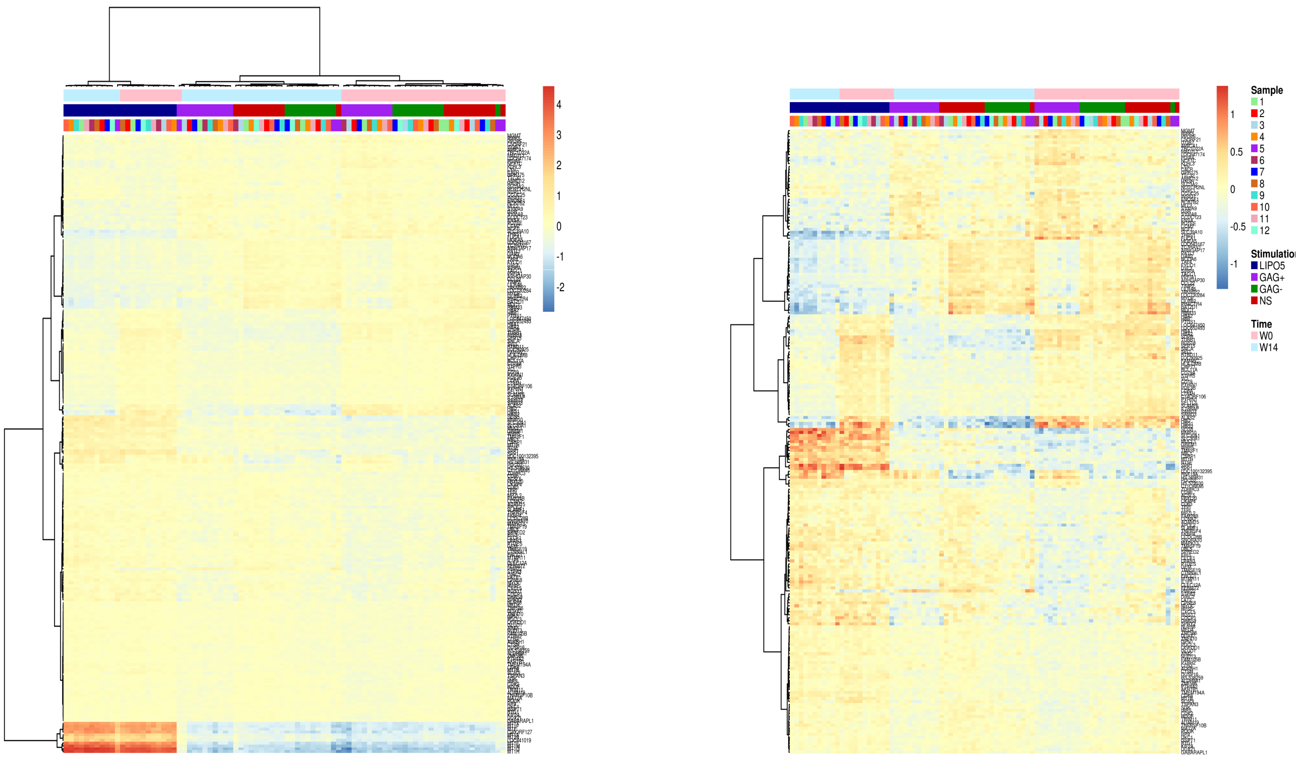

Figure 10: HIV study (transcriptomics data before and after vaccination). Hierarchical clustering (Euclidian distance and "ward" method aggregation) of the 220 genes selected with multilevel sPLS-DA (left). One cluster of genes over-expressed in LIPO5 has been removed (right). Samples are represented in columns and genes in rows.

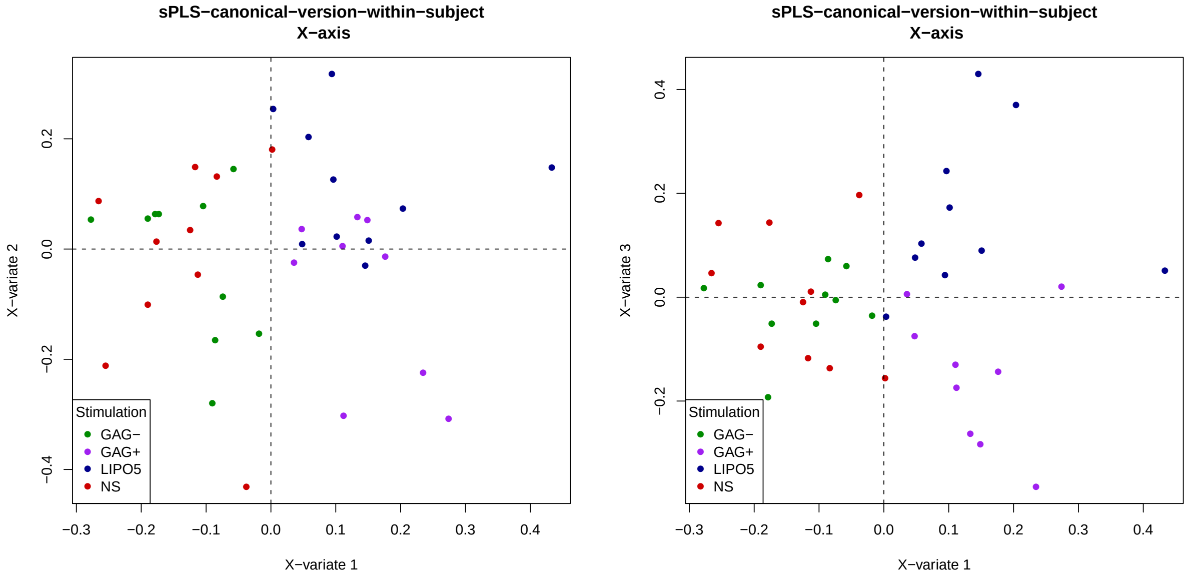

Figure 11: HIV study (integration of transcriptomics and cytokines data after vaccination). Sample representation with multilevel sPLS for dimensions 1-2 (left), 1-3 (right).
